# Evaluation of congenital and acquired heart diseases in a Spanish cohort of adults with Down syndrome

**DOI:** 10.1038/s41598-022-26918-0

**Published:** 2022-12-28

**Authors:** Laura Rabes, Laura Adán-Lirola, María del Pilar González-Molina, José María Galván-Román, Fernando Moldenhauer, Emilia Roy-Vallejo, Diego Real de Asúa

**Affiliations:** 1 Adult Down Syndrome Outpatient Unit, Department of Internal Medicine, Hospital Universitario de La Princesa, Diego de León 62, 28006 Madrid, Spain; 2 Down Syndrome Medical Interest Group-USA (DSMIG-USA), New York, USA

**Keywords:** Health care, Medical genetics

## Abstract

To describe congenital and acquired heart diseases in a Spanish cohort of adults with Down syndrome (DS), which could inform potential health recommendations for this population. Cross-sectional, observational study of adults with DS evaluated consecutively at a tertiary care, outpatient center between January 1 and December 31, 2019. The study population comprised 937 patients (51.8% men; median [IQR] age, 42 [18] years). An echocardiogram was available in the clinical chart of 420 patients (44.8%). The diagnosis of any form of heart disease was confirmed in 211 patients (22.5%): 101 (10.8%) had congenital heart defects, 80 (8.5%) simultaneous congenital and valvular heart diseases, and 30 (3.2%) isolated valvular heart disease. 111 patients (52.6% of those with congenital or valvular heart disease) had received corrective cardiac surgery. A total of 65 individuals were receiving medical management alone (30.8%), while 35 did not require any treatment because their cardiac disease was mild (16.6%). We found a high overall prevalence of heart disease in patients with DS, higher than previously reported for the pediatric population. Management of cardiovascular disease in adults with DS differs from that of the general population and should include universal echocardiography-based screening.

## Introduction

Down syndrome (DS), caused by total or partial trisomy of chromosome 21, is the most frequent chromosomal disease in live newborns^1^. Heart disease—congenital or acquired—is one of its most characteristic phenotypic traits of DS^2^. Congenital heart disease (CHD) has traditionally been considered one of the main causes of death in persons with DS^3,4^. However, recent decades have seen a significant increase in the life expectancy of this population, mainly thanks to early surgical repair of cardiac abnormalities^5–7^.

Unfortunately, the prevalence and outcome of heart disease in adults with DS remains largely unstudied^8,9^, and current health recommendations for this population are still based mainly on a translation of those for childhood CHD^10^.This gap of clinical data comprises our understanding of the prevalence, and impact of, classic cardiovascular risk factors, valvular heart disease, arrhythmias, and cardiovascular events, such as ischemic heart disease and stroke, and leads to healthcare recommendations which might not be appropriately tailored to the needs of the adult population with DS. Indeed, the Global Down Syndrome Foundation recently published clinical guidelines for the care of adults with DS in which they recommended periodic screening for cardiovascular risk factors such as diabetes and dyslipidemia and the follow-up of adults with a previous history of CHD^11,12^. However, the quality of these recommendations was considered low or very low, and none addressed the evaluation of heart disease that could have been acquired during adulthood. In fact, despite the confluence of multiple vascular risk factors, such as weight disorders and dyslipidemia, atherosclerosis (whether coronary or cerebral) seems almost nonexistent in adults with DS^13–15^.

Therefore, the objective of the present study was to determine the frequency of congenital and acquired heart diseases, including atherosclerotic cardiovascular risk factors and/or cardiovascular events in a Spanish cohort of 937 adults with DS to better inform potential healthcare recommendations for this population.

## Methods

### Study design

The study was performed in a tertiary care, university public hospital in Madrid, Spain. The center includes the only reference unit caring for adults with DS in the Autonomous Community of Madrid. Since it was formed in 2005, this unit has evaluated more than 1,500 patients, that is, approximately 40% of the adult population estimated to have DS in the Autonomous Community of Madrid.

We performed a cross-sectional and observational study of all adults with DS evaluated consecutively in the unit between January 1 and December 31, 2019. More recent data (2020) was not selected to avoid potential follow-up losses during the COVID-19 pandemic. No other selection criteria were applied. The study followed the STROBE checklist for cohort, case–control and observational studies (Suppl. File 1).

### Patient and public involvement

Adults with DS were not involved in the research’s design. They actively participated as study subjects in its conduct. Once the results have been published, they will be disseminated to participants and members of the Down syndrome community at large in an accessible format and language (suitable for lay readers) via dedicated websites of DS associations (Down España [https://www.sindromedown.net], Fundación Iberoamericana Down21[https://www.down21.org]) and research societies. (T21RS [https://www.t21rs.org], DSMIG-USA [https://dsmig-usa.org]).

### Tests and variables

We collected a series of variables for all participants, as follows: age (years); sex; place of residence (family home, institution, other); degree of disability, classified as independent (independent person or one requiring only minor supervision or verbal support to perform activities of daily living, with ability to read and write, and/or employed), requiring partial help (dependent in 1 or 2 of the following activities: showering, dressing, using stairs or urinary incontinence; with some ability to read and/or write, or attending a day center), and dependent (dependence in more than 2 of the previously mentioned areas, or dependence in feeding, walking, moving from one place to another, toileting or fecal incontinence, inability to read or write, unemployed, not attending a day center); and anthropometric variables such as weight (kg), height (m), and body mass index (kg/m^2^). Weight was measured on a calibrated scale, with the result rounded to the nearest 0.1 kg. Height was measured using a stadiometer and rounded to the nearest centimeter. We also recorded the following medical comorbid conditions: (a) classic cardiovascular risk factors, such as arterial hypertension according to the EHS/ESC 2018 criteria^16^, diabetes mellitus defined according to the ADA 2020 criteria^17^, dyslipidemia according to the ESC/EAS 2019 guidelines^18^, weight disorders (defined according to the criteria of the National Institutes of Health^19^), smoking, family history of premature cardiovascular disease^20^; (b) thyroid disease (hypo- or hyperthyroidism; (c) sleep apnea–hypopnea syndrome, (d) whether the participants had or had had CHD and the type (atrioventricular septal defect, ventricular septal defect, atrial septal defect, Fallot’s tetralogy, patent ductus arteriosus, hypertrophic cardiomyopathy, other), (e) presence and type of valvular disease (mitral regurgitation and/or stenosis, aortic regurgitation and/or stenosis, tricuspid regurgitation and/or stenosis, and pulmonary regurgitation and/or stenosis), as well as (f) presence of ischemic heart disease and/or stroke, together with the underlying cause of the latter (cardioembolic, atherothrombotic, Moya-Moya disease, congenital stenosis of cerebral arteries, unknown). We acknowledge that most valvular diseases in adults with DS, even those appearing after the first decades of life, could also be considered congenital in nature (i.e., non-ischemic, non-rheumatic diseases). For the purpose of the study, to allow a structured classification of findings, we have nonetheless preserved the distinction between CHD (referring to septal defects and other anomalies) and valvular diseases. Those cases in which the valvular lesion was part of a greater syndrome (such as Fallot’s tetralogy), were included in the CHD group, and not in the valvular disease group. Finally, we collected variables associated with the diagnosis and treatment of heart disease: year of diagnosis; date of the last known transthoracic echocardiogram (years); treatment (none, medical -i.e., pharmacological- treatment, owing to absence of indication for surgery, medical management owing to delayed diagnosis, medical treatment owing to the degree of disability, surgical management and/or both medical and surgical treatment); and whether heart disease had resolved after surgery.

### Statistical analysis

Categorical data are expressed as frequencies (percentages), and quantitative variables as mean (standard deviation, SD) or median (Interquartile range, IQR). The χ^2^ test or the Fisher exact test were used for comparisons involving qualitative variables and the *t* test was used for quantitative variables. Statistical significance was set at *p *< 0.05 (2-tailed). We performed all data analyses using SPSS (Version 20.0.0, IBM Corp., Armonk, NY, USA).

### Ethical aspects

The study was carried out in line with the principles of the Declaration of Helsinki^21^ and current good clinical practice guidelines. The institutional review board at the Hospital Universitario de La Princesa, Madrid, approved the study and the exemption from requesting informed consent owing to the absence of an intervention in the study population and its retrospective and anonymous nature, where patients were only identified by their age (registry no. 3191/2019). Data confidentiality was guaranteed in line with current Spanish legislation (Ley orgánica 3/2018, de 5 diciembre, de protección de datos personales y garantía de los derechos digitales).

## Results

### Baseline sociodemographic characteristics

A total of 937 patients were included in the study (485 men [51.8%]; median [IQR] age, 42^18^ years, age range 17–67 years). Mild intellectual disability was recorded in 469 (49.9%), moderate intellectual disability in 312 (33.3%), and severe intellectual disability in 139 (14.8%), while this information was absent in 18 cases (1.9%). During the study period, 682 patients were living in their family home (72.8%).

### Prevalence of cardiovascular risk factors and events in adults with Down syndrome

The most prevalent cardiovascular risk factor was weight disorders (328 patients [35% of the total sample, 64% of all patients with recent data on weight and height available in the clinical history]), and the median BMI was 27.7 (7.2) kg/m^2^, followed by dyslipidemia (80 patients [8.5%]). Primary hypothyroidism and obstructive sleep apnea were also prevalent in the cohort (527 patients [56.2%] and 173 patients [18.5%], respectively). The prevalence of other cardiovascular risk factors is summarized in Table 1.

**Table 1:** Prevalence of cardiovascular risk factors among Spanish adults with Down syndrome.

	Study population (n = 937)
Age (IQR, in years)	42 (18)
Sex (% males)	485 (51.8)
Family history of early cardiovascular disease	39 (4.2)
Body mass index (IQR, in kg/m^2^)	27.7 (7.2)
Weight disorders (%)	Low weight: 6 (0.6)
	Normal weight: 178 (19)
	Overweight: 175 (18.7)
	Grade 1 Obesity: 90 (9.6)
	Grade 2 Obesity: 50 (5.3)
	Grade 3 Obesity: 13 (1.4)
Arterial hypertension (%)	3 (0.3)
Diabetes mellitus (including types 1 and 2, in %)	19 (2)
Dyslipidemia (%)	80 (8.5)
Smoking (%)	2 (0.2)
Thyroid function	Euthyroidism: 390 (41.6)
	Hypothyroidism: 527 (56.2)
	Hyperthyroidism: 14 (1.5)
Obstructive sleep apnea	173 (18.5)

Data are presented as n (%) or median (IQR).

Only 9 cases (1%) of cardiovascular events were recorded, all of which were strokes. The most frequent causes thereof were cardiac embolism (4 patients) and Moya-Moya disease (4 patients). The nature of the event remained undetermined in 1 case. No patients in the sample had had ischemic heart disease.

### Congenital and valvular heart disease in adults with Down syndrome: prevalence and treatment options

**Figure 1:**
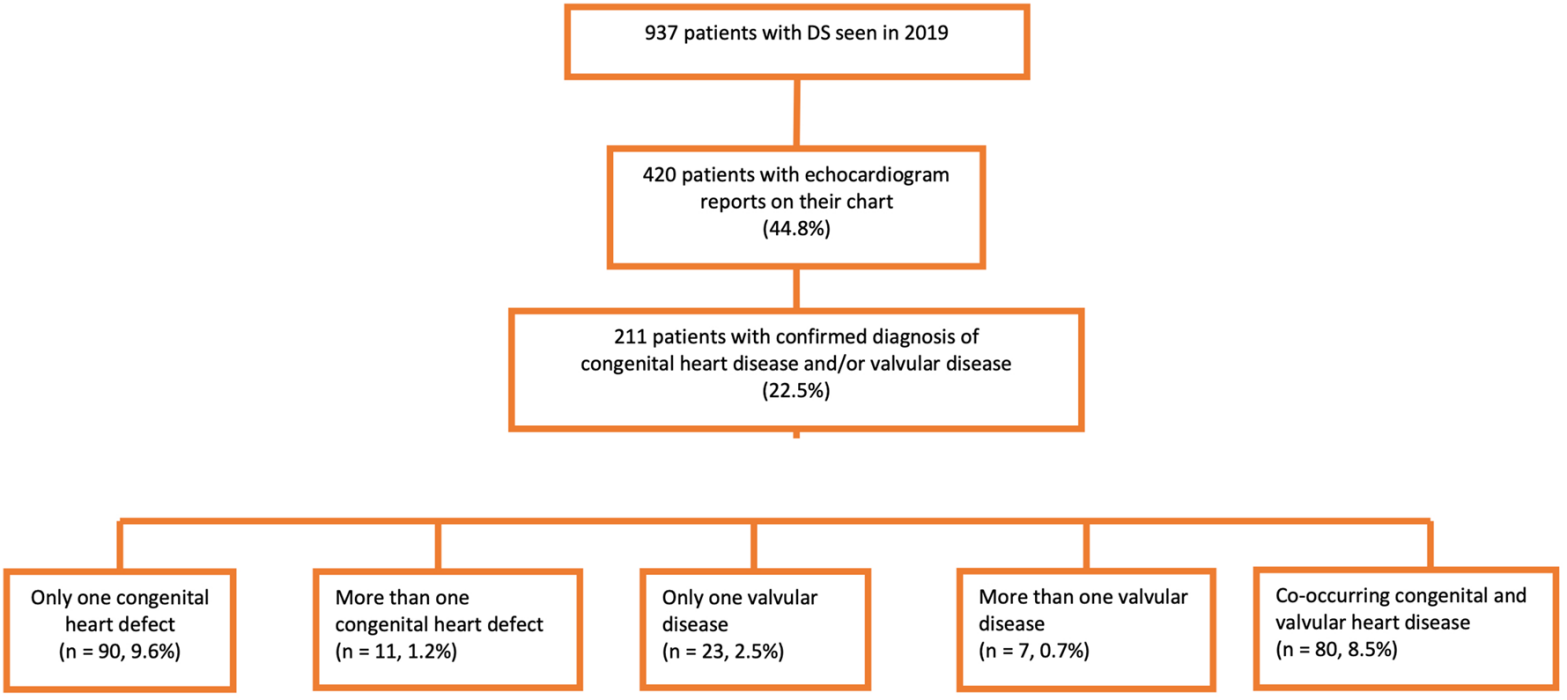
Diagnostic categories of congenital and valvular heart disease in Spanish adults with Down syndrome (data are presented as n (%)).

An echocardiogram report was available in the clinical chart of 420 patients (44.8% of the study sample). The diagnosis of CHD or valvular disease was confirmed in 211 patients (50.2%, 22.5% of our study sample). A total of 101 patients had congenital heart defects (24% of those with an echocardiogram, 10.8% of the study sample), and a further 80 presented simultaneous congenital heart and valvular heart disease (19% of those with an echocardiogram, 8.5% of the study sample). Isolated valvular heart disease was observed in 30 patients with DS (7.1% of those with an echocardiogram, 3.2% of the study sample). The distribution of patients between diagnostic categories is summarized in Fig. 1.

Ventricular septal defects were the most frequent form of CHD in adults with DS (59 patients, 32.6% of adults with CHD), followed by atrial septal defects (50 patients [27.6%]). Of these, ostium primum defects were more prevalent than ostium secundum atrial septal defects (32 patients vs. 18 [17.7% vs 9.9%] respectively). Atrioventricular heart defects (48, 26.5%) and persistent ductus arteriosus (33 adults, 18.2%) were also frequently detected in our sample. The prevalence of other forms of CHD is presented in Table 2.

**Table 2:** Prevalence of congenital heart diseases in Spanish adults with Down syndrome.

Congenital heart disease	n	Prevalence among patients with congenital heart disease (%)	Prevalence among patients with an echocardiogram available (%)	Full sample prevalence (%)
Ventricular septal defect	59	32.6	14.0	6.3
Atrioventricular septal defect	48	26.5	11.4	5.1
Persistent ductus arteriosus	33	18.2	7.9	3.5
Ostium primum	32	17.7	7.6	3.4
Ostium secundum	18	9.9	4.3	1.9
Tetralogy of fallot	10	5.5	2.4	1.1
Hypertrophic cardiomyopathy	5	2.8	1.2	0.5
Other	14	7.7	3.3	1.5

Mitral regurgitation was the most frequent valvular disease diagnosed in our sample (59 patients, 53.6% of those with valvular disease). Tricuspid regurgitation (36 patients, 32.7%) and aortic regurgitation (33 patients, 30%) were the second and third most prevalent valvular lesions, respectively. Valvular stenoses were very uncommon in our sample (Table 3).

**Table 3:** Prevalence of valvular heart disease in Spanish adults with Down syndrome.

Valvular disease	n	Prevalence among patients with valvular disease (%)	Prevalence among patients with echocardiogram (%)	Full sample prevalence (%)
Mitral regurgitation	59	53.6	14.0	6.3
Tricuspid regurgitation	36	32.7	8.6	3.8
Aortic regurgitation	33	30.0	7.9	3.5
Pulmonary regurgitation	15	13.6	3.6	1.6
Pulmonary stenosis	11	10	2.6	1.2
Tricuspid stenosis	4	3.6	1	0.4
Aortic stenosis	2	1.8	0.5	0.2
Mitral stenosis	0	0	0	0
Unknown	1	0.9	0.2	0.1

Corrective cardiac surgery had been the treatment of choice for 111 patients with DS (52.6% of the subsample with congenital or valvular heart disease). All interventions took place during childhood. A further 65 individuals were receiving medical management alone (30.8%), while 35 (16.6%) did not require any treatment at all due to the mild nature of their defects. Regardless of the treatment modality, only 9 patients with DS had had a documented episode of heart failure in their clinical charts.

## Discussion

More than half of the adult patients with DS for whom imaging data were available had CHD or valvular heart disease. Of these 211 patients, almost half had at least one congenital heart defect (101 adults, 47.8%) and a further third had coexistent valvular and non-valvular defects (80 patients, 37.9%). The prevalence of classic cardiovascular risk factors, such as hypertension and diabetes, or of cardiovascular events was very low, even despite the high prevalence of weight disorders in adults with DS in our sample.

We observed considerable agreement between our results and those formerly published for the pediatric population. In terms of CHD types, Freeman et al. also observed in a broad pediatric cohort in the United States that ventricular septal defect was the most prevalent heart disease, affecting more than 4 in every 10 children with DS and CHD^22^. This type of heart disease was followed by atrial septal defects (43%) and defects of the atrioventricular canal (39%). Albeit with minor differences in the prevalence of the different types of septal defect, our results also mirror other American and in European studies^23–25^. The explanation for these slight geographical differences in the prevalence of heart disease is unclear, and both genetic and environmental causes have been posited^22^.

Noteworthy, we observed a global prevalence of heart disease among that was somewhat higher than previously described in the pediatric population^22–25^. There are 2 possible explanations for this finding. First, during neonatal or childhood screening, some cases may not have been detected, especially if the initial defect was very mild. In addition, this group of adults with childhood CHD may have developed new-onset valvular disease during adulthood. Some adults may have gone undiagnosed simply because of the absence of proper screening during childhood^9^.

These results enable us to draw conclusions worthy of translation into clinical practice. The high prevalence of heart disease in adults with DS highlights the need to recommend echocardiogram-based screening in future clinical practice guidelines. While this hypothesis has already been put forward by several authors^11,26^, sufficient supporting evidence had been lacking.

We are aware that this recommendation may draw criticism. Despite the high prevalence of heart disease in adults with DS, we acknowledge that the clinical impact of future, screening-based findings during adulthood is uncertain. After all, one in every six adults with DS in our sample did not require specific treatment for their heart disease owing to the mild severity of their condition. This finding (and the low prevalence of heart failure in our sample) may partly derive from the fact that a cardiological evaluation was not routinely performed on all subjects with heart disease, and thus the true prevalence of heart failure might as well be underestimated. However, these considerations do not diminish interest in increasing diagnostic efforts. The increasing life expectancy of adults with DS makes it difficult to predict progress over decades in cases of mild heart disease in childhood without solid data. In order to perform longitudinal studies, we need to start with a robust knowledge of the prevalence of the problem. This will only be possible if we can first extend the recommendation for imaging-based screening to the whole adult population with DS.

We recorded a very low prevalence of classic cardiovascular risk factors and cardiovascular events in adults with DS. In classic studies, Murdoch et al. and Yla-Herttüala et al. recorded the absence of atheromatous plaque in autopsies performed in institutionalized adults with DS^14,15^. In clinical practice, the prevalence of coronary events has been shown to be lower in adults with DS than in the general population^26,27^. The difference in distribution of vascular risk factors has been put forward as an explanation. While weight disorders are more prevalent in adults with DS than in the general population, these patients are, in contrast, less likely to be hypertensive, diabetic, or smokers. A key aspect of this phenotype of protection against atherosclerosis is the absence of arterial hypertension, which seems to be related to the poorer development of the sympathetic nervous system^28,29^.

Protection against atherosclerosis not only affects the coronary territory, but also extends to the cerebral vascular territory. While the prevalence of stroke we recorded is higher than in the general population, this increased risk cannot be attributed to atherosclerosis, but rather to cardioembolic mechanisms or Moya-Moya disease. These findings neither support recommendations to early detect and treat vascular risk factors, such as dyslipidemia, not to extend the use of 10-year vascular risk calculators, which have proven useful in the general population, to the persons with DS^11^. In fact, the finding of a greater proportion of cardioembolic strokes compared to the general population further reinforces our recommendation to extend echocardiography-based screening to adults with DS.

Our study is subject to a series of limitations. First, the present number of patients with echocardiographic data, and the lack of knowledge of the motives for pursuing an echocardiographic evaluation could lead to underestimate the true prevalence of heart disease. However, this does not weaken our conclusions. If our estimated prevalence were to increase, the need to extend imaging-based screening to all persons with DS can only grow. Second, the cross-sectional design of the study prevented us from evaluating the incidence of new events over time. The strengths of our study include its large sample and exhaustive collection of clinical variables. The high consistency between current results and those of previous pediatric studies also serve as an indirect proof of their external validity. We were unable to determine whether the current heart defects found on echocardiography were congenital unrepaired defects or residual post-surgical defects. Thus, we reckon that some of the valvular diseases identified in our study could have derived from sequels of prior heart surgeries. Although our current methodology does not allow for an appropriate differentiation of their potential etiology, the goal of the study was to determine their prevalence to draw conclusions that might guide the need for a widened screen of heart diseases in adults with DS, an objective which has been achieved.

Our study revealed an overall prevalence of heart disease in adults with DS and known echocardiographic data that was slightly higher than that reported for the pediatric population. Atherothrombotic events in adults with DS were practically nonexistent. Therefore, we believe that our results should lead us to reconsider current recommendations in guidelines on the care of adults with DS: management of cardiovascular risk factors should clearly differ from that of the general population and all adults with DS should undergo echocardiography-based screening.

## Supplementary Information

Supplementary Information 1.

## Data Availability

A fully anonimized version of the datasets used/analysed during the current study is available from the corresponding author on reasonable request.
